# Is the colon mucosa affected by ten days of gastric restriction in an animal model?^1^

**DOI:** 10.1590/s0102-865020190060000010

**Published:** 2019-08-19

**Authors:** Flávia Emi Akamatsu, Luiz Gustavo Fontes, Ana Maria Itezerote, Samir Saleh, Walcy Paganelli Rosolia Teodoro, Everson Artifon, Flávio Hojaij, Mauro Andrade, José Aires Pereira, Carlos Augusto Real Martinez, Alfredo Luiz Jacomo

**Affiliations:** I PhD, Department of Surgery, Laboratory of Medical Research 02, Division of Human Structural Topography, Faculty of Medicine, Universidade de São Paulo (FMUSP), Brazil. Design, intellectual and scientific content of the study; manuscript writing.; II Graduate student, Division of Human Structural Topography, FMUSP, Sao Paulo-SP, Brazil. Acquisition of data, technical procedures.; III PhD, Department of Surgery, Laboratory of Medical Research 02, Division of Human Structural Topography, FMUSP, Sao Paulo-SP, Brazil. Acquisition of data, technical procedures.; IV PhD, Department of Surgery, Laboratory of Medical Research 02, Division of Human Structural Topography, FMUSP, Sao Paulo-SP, Brazil. Acquisition of data, technical procedures.; V PhD, Department of Rheumatology, Laboratory of Medical Research-Medical Clinica, FMUSP, Sao Paulo-SP, Brazil. Technical procedures, interpretation of data.; VI PhD, Department of Surgery, Division of Human Structural Topography, FMUSP, Sao Paulo-SP, Brazil. Statistical analyses, critical revision.; VII PhD. Department of Surgery Medicine, Laboratory of Medical Research 02, FMUSP, Sao Paulo-SP, Brazil. Technical procedures, interpretation of data.; VIII PhD, Department of Surgery, Laboratory of Medical Research 02, Division of Human Structural Topography, FMUSP, Sao Paulo-SP, Brazil. Interpretation of data, manuscript writing, critical revision.; IX PhD, Department of Health Science, Universidade São Francisco (USF), Bragança Paulista-SP, Brazil. Technical procedures, histological examinations.; X PhD, Department of Health Science, USF, Bragança Paulista-SP, Brazil. Conception of the study, analysis and interpretation of data.; XI PhD, Department of Surgery, Laboratory of Medical Research 02, Division of Human Structural Topography, FMUSP, Sao Paulo-SP, Brazil. Conception, intellectual and scientific content of the study, critical revision.

**Keywords:** Models, animal, Colon, Mucins, Rats

## Abstract

**Purpose:**

To identify whether the colon mucosa is affected by ten days of gastric restriction in an animal model.

**Methods:**

An experimental model of gastric restriction was devised using rats. The animals were submitted to surgical gastrostomy, and a cylindrical loofah was inserted into the stomach. We studied 30 adult male Wistar rats divided into three groups: the stomach restriction group (R10); the sham group (S10), which underwent the same procedure except for the loofah insertion; and the control group (C10). The expression of neutral and acid mucins was evaluated using histochemical techniques. Goblet cells and protein content were compared between groups using generalized estimation equations (GEEs). Bonferroni’s multiple comparison was applied to identify differences between the groups. All tests considered a 5% significance level.

**Results:**

There was an increased expression of neutral mucins, acid mucins and goblet cells in the R10 group. Collagen was also enhanced in the R10 group.

**Conclusion:**

The colon mucosa is affected by ten days of gastric restriction in an animal model, increasing neutral mucins, acid mucins and collagen content with trophic maintenance.

## Introduction

Obesity is a global epidemic^1^, and in adulthood, it is a major risk factor for the world’s leading causes of poor health and early death, including cardiovascular disease, several common cancers, diabetes and osteoarthritis^2^. The burden of disease generated from the high prevalence and consequences of obesity makes it a true global public health concern^3^. The only proven long-term treatment for severe obesity on a population level is the surgical modification of the gastrointestinal anatomy to induce weight loss, termed bariatric surgery^4^. Bariatric surgery, also called weight loss surgery or metabolic surgery, was introduced 50 years ago to provide drastic weight loss in morbidly obese patients^5,6^, and its use is currently increasing to treat patients with high adiposity, or occasionally for metabolic benefits^7^. The most successful types of bariatric surgery involve limiting the absorption of nutrients (malabsorption), reducing the size of the stomach to decrease the total nutrient intake (restriction), and reducing hunger and satiety by altering gut hormones (metabolic)^3^. Sleeve gastrectomy (SG), gastric bypass, gastric banding, and gastric plication (GP) are the most common procedures used to treat obesity^8^. However, recent studies have suggested serious complications that may result from weight reduction surgery, such as nutritional deficiencies^9-13^, the acceleration of nephropathy^14^, modification of the hippocampus cytoarchitecture^15^, changes in gene expression for subcutaneous fat and arcuate hypothalamic nucleus^16^and modifications in bone metabolism, accelerating the process of osteoporosis^17^. A body weight loss of 15% or more is associated with the loss of 20% of the body’s protein^18^. Recently, authors have reported constipation after bariatric surgery with diet, due to decreased bowel motility and prolonged bowel movement^19^. Restrictive diets and bariatric surgery related to nutrient deficiency rather than weight loss surgery reduce the microbial abundance and promote changes in the microbial composition that could have long-term, detrimental effects on the colon^20^. Goblet cells reside throughout the length of the small and large intestine and are responsible for the production and maintenance of the protective mucus blanket by synthesizing and secreting high-molecular-weight glycoproteins known as mucins. The tissue content and pattern of mucin expression in goblet cells change in patients with colorectal cancer, ulcerative colitis, intestinal infections, inflammatory bowel disease (IBD), cystic fibrosis (CF), and diversion colitis^21-23^. Disruption in the intestinal homeostasis results in a defective mucus barrier with increased permeability, which results in inflammation and injury of the intestinal mucosal cells^24,25^. Studies in experimental models have demonstrated that a deficiency in the supply of short-chain fatty acids (SCFAs) to the colon, devoid of transit, can modify proteins related to the intercellular junction systems, allowing the development of exclusion colitis^22,26,27^. SCFAs influence colonic health through various mechanisms. In vitro and ex vivo studies have shown that SCFAs have anti-inflammatory and anticarcinogenic effects, play an important role in maintaining metabolic homeostasis in colonocytes, and protect colonocytes from external harm^28,29^_._

The effect on the colon caused by malnutrition due to gastric restriction surgeries in the stomach has not yet been studied. The objective of the present study is to evaluate and quantify the acid and neutral mucin contents, as well as the number of goblet cells in the colon and their respective crypts, of rats submitted to gastric restriction for 10 days. We also intend to correlate any changes found with acute weight loss, with the aim of contributing to the effectiveness and safety of this therapy that has been increasing worldwide.

## Methods

### Animals and experimental protocol

This study was approved by the Ethics Committee for the Analysis of Research Projects (Protocol 065/13). All animals received humane care in compliance with the experimental protocols of the Ethical Principles in Animal Experiments adopted by the Brazilian Association of Animal Testing.

Thirty adult male *Rattus norvegicus albinus* Wistar animals weighing 250 to 300 g were housed in the Universidade de São Paulo, School of Medicine’s facilities. Five animals were housed per cage and were given food and purified tap water *ad libitum*. The animals were operated on in the Center for Study and Research in Surgery (CEPEC), Department of Urology of the Faculty of Medicine. The animals were divided into three groups: the stomach restriction group (R10), the sham group (S10), which underwent the same procedure except for the loofah insertion, and the control group (C10). Animals were fed and kept in separate cages. They were weighed every other day until being sacrificed on day 10.

### Surgery to promote gastric reduction

To reduce the gastric capacity and cause significant weight loss in rats without developing anemia, a surgical procedure was performed, similar to that described by Tolosa *et al*.^30^. After 12 hours of fasting, the rats were anesthetized by isoflurane 4% vaporization. Anesthesia was continued through a mask with 1.5-3% isoflurane inspired fraction, and the rats were submitted to median laparotomy and gastrostomy in the greater curvature of the stomach for the implantation of a cylindrical Luffa bush (loofah; 1.5 cm in diameter) made of cylindrical Luffa (an experimental phytobezoar). The gastrostomy was closed with continuous mononylon suture yarn 6.0, and the abdominal wall was closed in two planes: the aponeurosis, with 4.0 mononylon suture yarn, and the skin, with 3.0 plain cotton stitches. The animals in the sham group were exposed to the same surgical procedure described above but were not submitted to phytobezoar implantation. Tramadol hydrochloride (20-40 mg/kg) was administered intraperitoneally, with a frequency of at least 12 hours for 5 days^31^.

### Experimental groups

On day 1, the animals were divided into three groups of 10 animals: one group underwent surgery for stomach restriction (R10), with both the sham controls (S10) and normal controls (C10). Each animal was weighed every two days until the day of euthanasia.

### Euthanasia and material collection

After the experimental period, the animals were euthanized with carbon dioxide (CO_2_). This gas is lethal because it causes depression of the central nervous system. The animals were kept in the chamber for more than 10 minutes to confirm their death^32^. Two colon samples were collected 2 cm from the cecum and were individually packed into containers. Samples were submitted to routine histological processing for Hematoxylin-Eosin (HE), Alcian Blue (AB), Periodic Acid-Schiff (PAS), High Iron Diamine (HID-AB) and Picrosirius Red staining.

### Histological morphometric analysis

The Zeiss Microscope Imager.A2 was used for the analysis of the slides with HE. The images were captured by a camera coupled with the AxioVision Release 4.8.1 program. The entire length of the crypts of the colon mucosa was measured and the goblet cells were counted, with three crypts per field. We used six fields per sheet from the control, sham and experimental groups, which were analyzed at a magnification of x400. We also analyzed the inflammatory infiltration in the colon mucosa. Polymorphonuclear and lymphomononuclear cell counts were performed in three areas of the inflammatory infiltration. Inflammatory cell counts were performed in a fraction of the inflammatory cell area. Polymorphonuclear and lymphomononuclear cells were counted in six fields of the area of inflammatory infiltration. The number of inflammatory cells was expressed by dividing the number of cells by the area of the infiltration.

Mucins were quantified in the colon of the control, sham and experimental groups using a computer-assisted image analysis. Briefly, an image analysis system consisting of an Olympus camera mounted on a microscope sent the images to a monitor using a computer-controlled (Pentium 1330 MHz) digitizing system (Oculus TCX, Coreco Inc., St. Laurent, Quebec, Canada). The images were processed by *Image-Pro Plus 7.0* software (Media Cybernetics, Inc., Bethesda, MD, USA). For each lamina, three crypts per field were measured, and six fields per sheet from the control, sham and experimental groups were analyzed at a magnification of x400. The mucin content in this compartment was expressed as the number of mucins divided by the total area studied. The final results were expressed as the number of mucins per total area. The measure of the tissue content of Picrosirius Red was analyzed with the same methodology as described above utilizing a polarized light.

### Statistical analysis

Weight, goblet and inflammatory cell and mucin tissue expression (PAS, AB, and HID-AB) were presented by groups as the mean, median, minimum and maximum.

Between-group comparisons were made using generalized estimation equations (GEE) for data with a normal distribution, as well as an identity link function, supposing an exchangeable correlation matrix between fields^33^. Inflammatory cells were compared between groups and types using GEE with Poisson distribution due to cell absence. Bonferroni’s multiple comparison was applied to identify differences between groups^34^. For weight, a multiple-group comparison was performed using an analysis of variance (ANOVA) followed by Bonferroni’s post hoc test. To verify the correlation between the weight and height of the crypt and the cell quantity, Spearman’s correlation was calculated^35^. To perform the analysis, IBM-SPSS software for Windows version 22 was used, and the data were tabulated using Microsoft Excel 2013 software. All tests were carried out with a significance level of 5%.

## Results

The statistical analysis showed that the weight of the restricted group decreased significantly (by 9%) in the first three days, and then increased (by 6.2% of the weight of the third day), which was conserved until the tenth day. Up to the 10th day there was a loss of approximately 4% of the initial weight. The weight of the sham group increased significantly from the first day to the tenth day, with an increase of 5.2% of the initial weight. The weight of the control group increased significantly from the first day to the tenth day, with an increase of 11% of the initial weight (Fig. 1).

**Figure:**
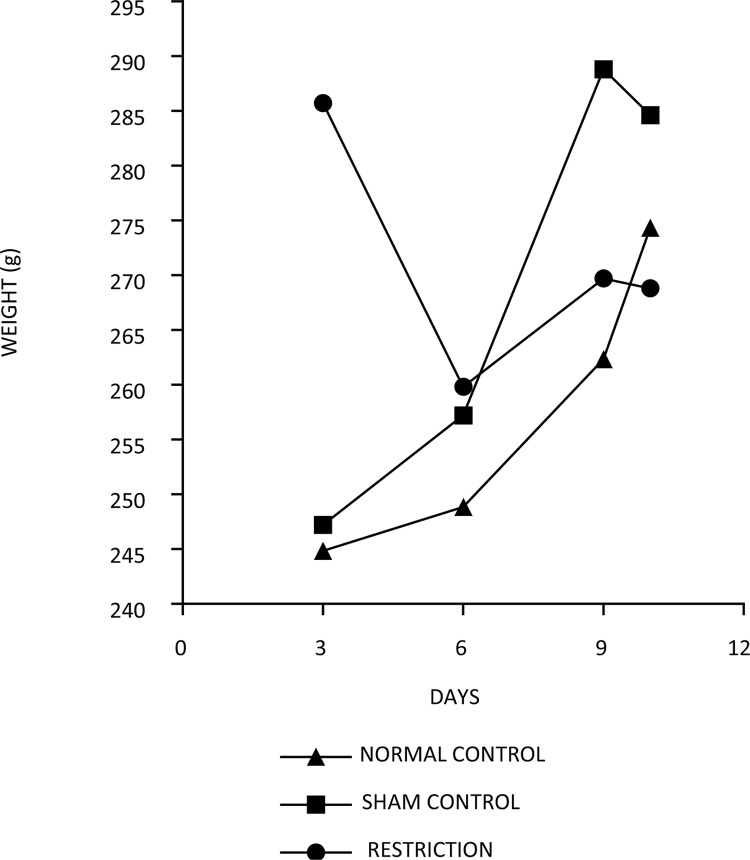
Figure 1 – The evolution of weight over 10 days in the normal, sham and restriction groups.

In the between-group comparisons of polymorphonuclear and lymphomononuclear cells, the polymorphonuclear cells and lymphocytes behaved similarly between the groups; there was a significant difference in the cell types, with pmn > lymphocytes in all groups, and there was a significant difference between the groups independent of cell type (Fig. 2). In HE, it was found that after 10 days of gastric restriction, the height of the crypts in general decreased when compared to the sham and control animals (p<0.05).

**Figure:**
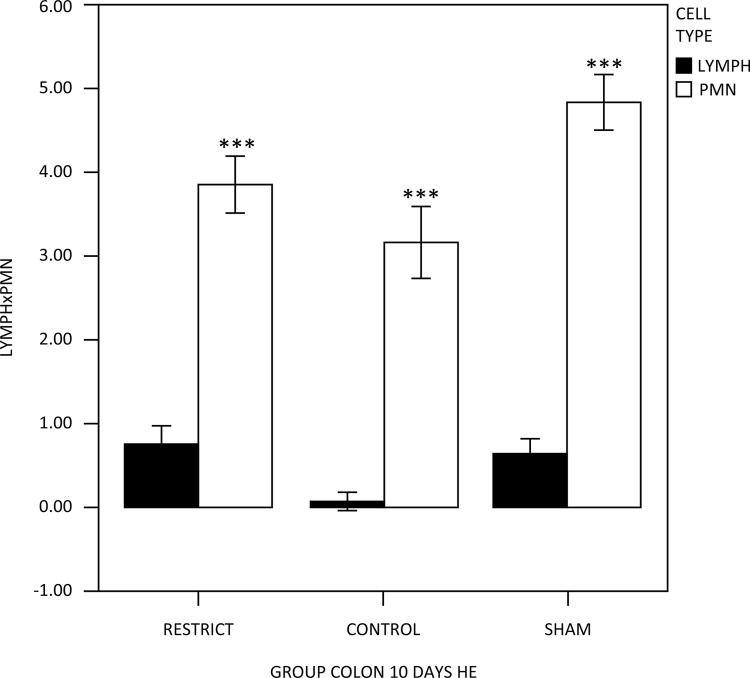
Figure 2 – Polymorphonuclear and lymphomononuclear cell counts showing similarly between the groups; a significant difference in the cell types, with pmn > lymphocytes in all groups (p<0.001***).

**Figure 4:**
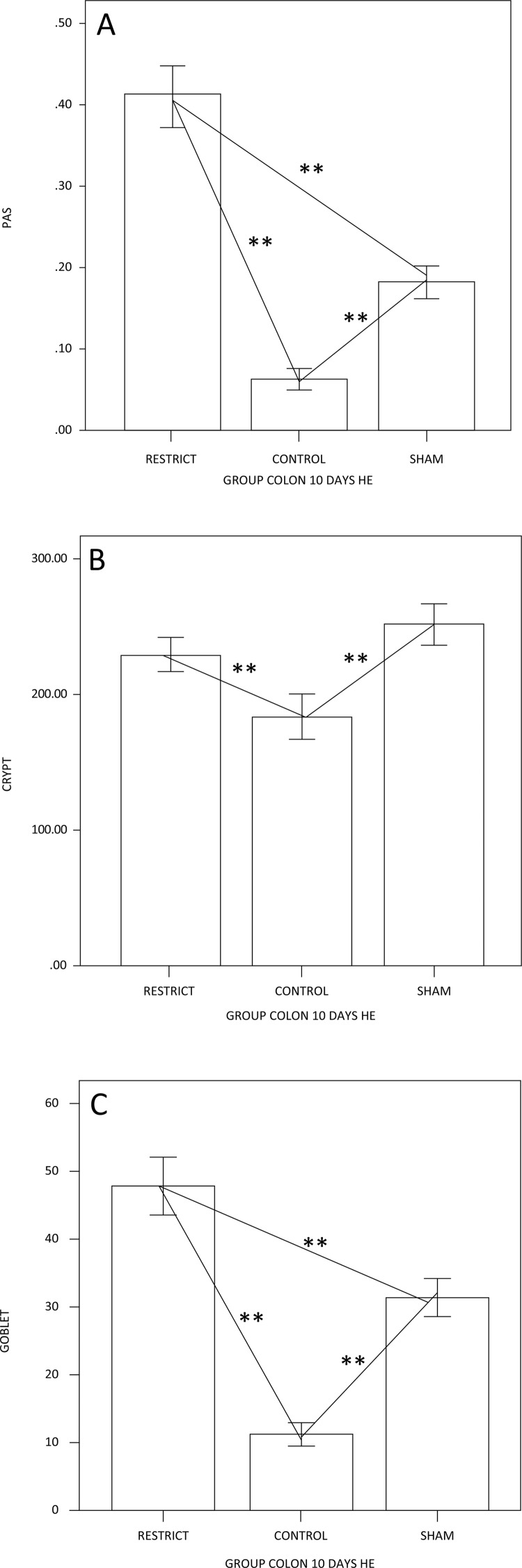
– (A) Semiquantification of neutral mucins; the restriction group has more neutral mucins than the other groups. (B) The difference in crypt height is only related to the control group; the goblet cells are similar to A (p<0.05**).

The number of goblet cells showed no difference compared to the sham group (Figs. 3 and 7). There was a trend of maintaining trophism for 10 days observed in HE (Fig. 3), with an increase in neutral mucins and their cells and an increase in acid mucins (sulfomucins) and their respective crypts (Figs. 4 and 5). There were no differences in sialomucins or sulfomucins in the restricted group compared to the sham group, as well as height and the number of cells compared to the control group. We found a significant increase in total collagen in the restricted group compared to the sham and control groups (Figs. 6 and 7).

**Figure:**
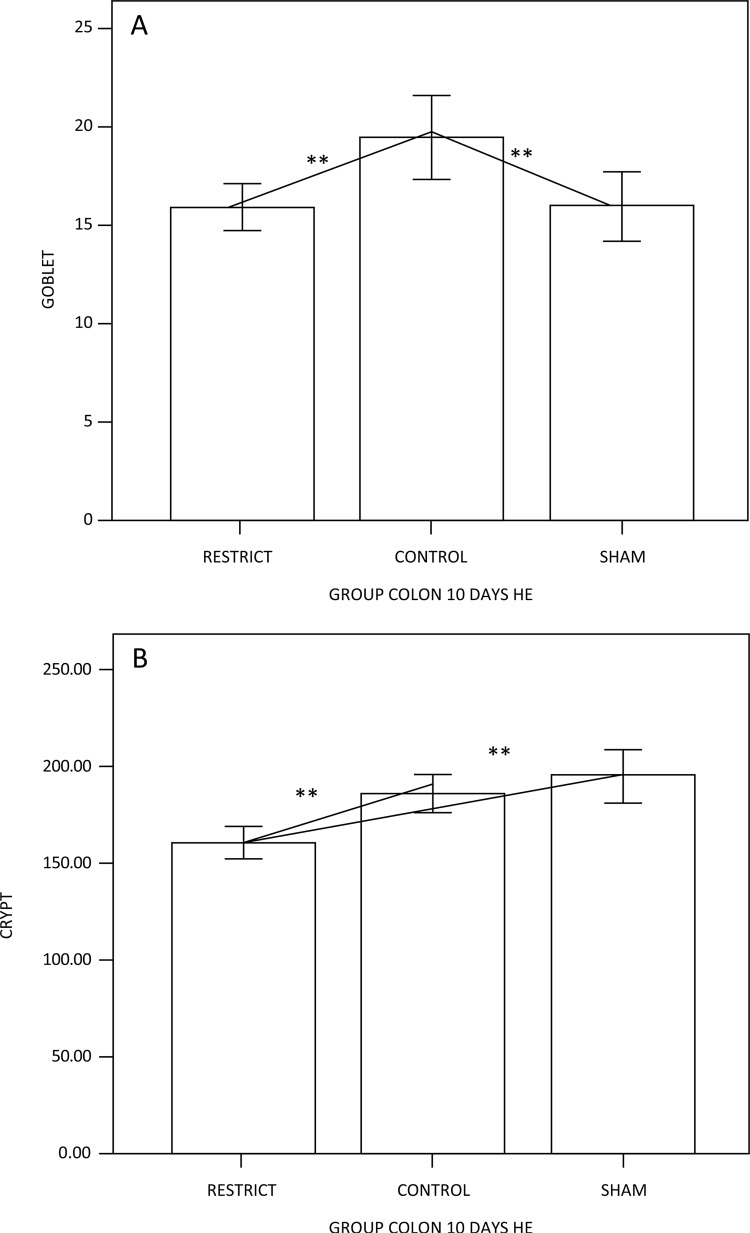
Figure 3 – (A) Goblet cell counts, showing a difference between the groups; the restricted and sham groups have fewer goblet cells than the control group. (B) Crypt height, showing the decrease in height of the restrict group when compared to the sham and control animals (p<0.05**).

**Figure:**
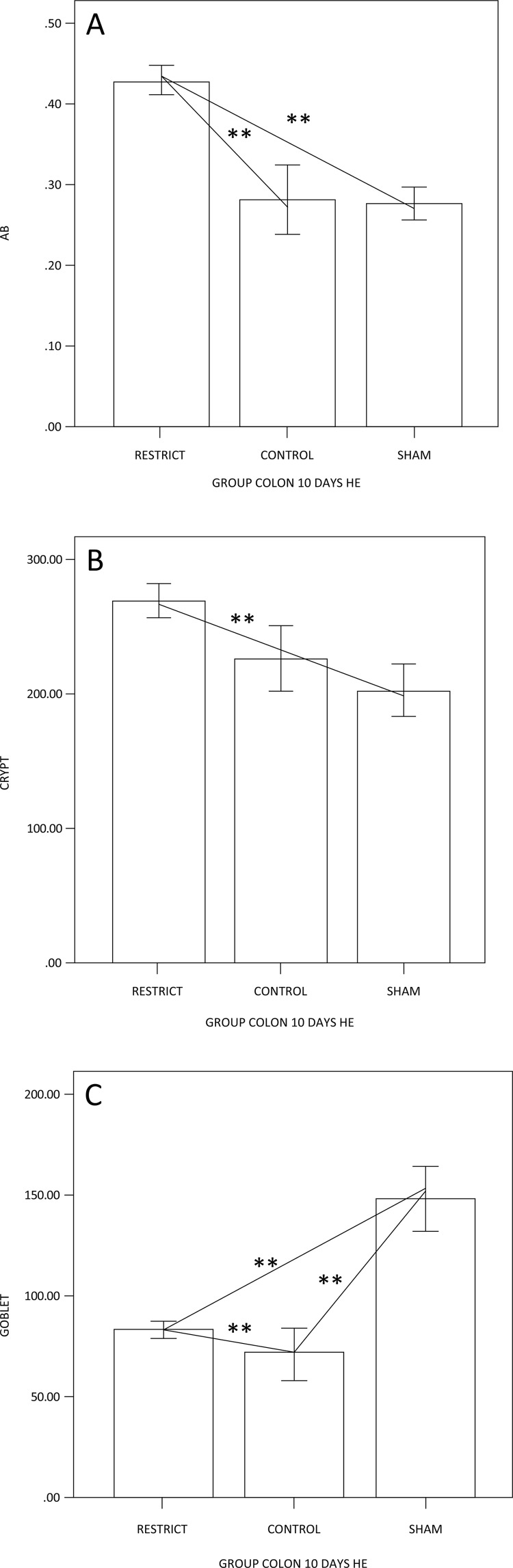
Figure 5 – (A) Semiquantification of AB acid mucins; the restriction group has more acid mucins than the other groups. (B) The difference in crypt height is only related to the restricted and sham groups; the goblet cells show that the restrict group is enhanced only in relation to the control group (p<0.05**).

**Figure:**
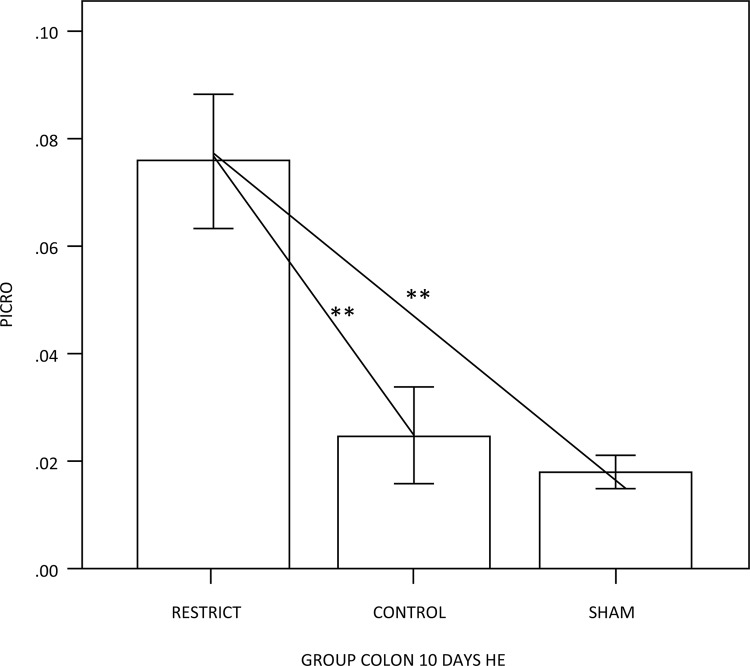
Figure 7 – Goblet cells x400: (A-C) HE; (D-F) PAS; (G-H) AB; (J-L) Sirius Red. Not much difference can be observed between the crypts in A, B and C. There are more goblet cells in D and G. In J-L, the restrict group has more collagen than the control and sham groups.

**Figure:**
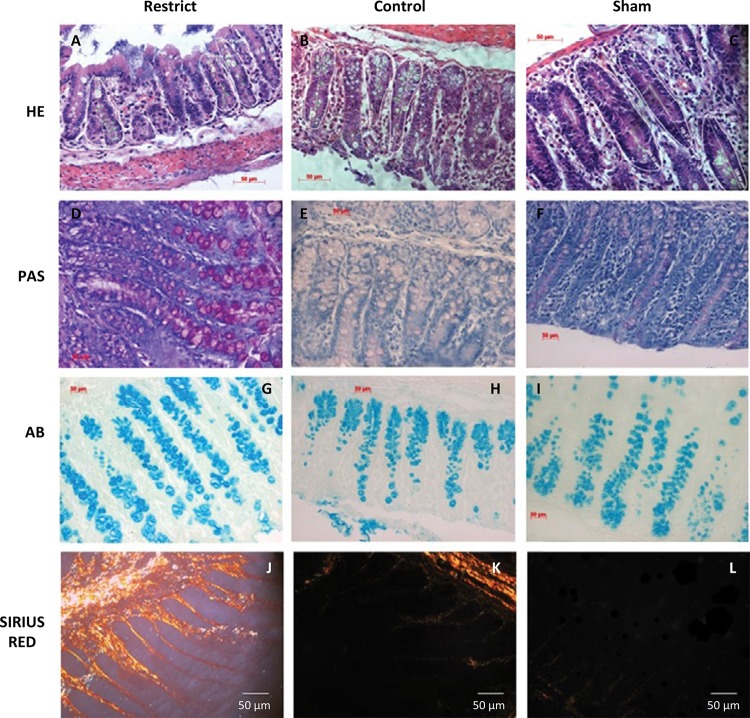
Figure 6 – Semiquantification of total collagen: collagen was enhanced in the restrict group compared to the control and sham groups (p<0.05**).

There was no correlation of weight with the goblet cells stained by PAS, AB, or HID-AB (p = 0.051), and although not significant, these data indicate that for the height of the crypt controlled for PAS, the weight and height of the crypt increase.

There was a correlation between control weight and polymorphonuclear control cells; when polymorphonuclear cells increased, weight decreased.

## Discussion

Experimental bariatric surgery was performed to prevent weight gain. Studies in animals have shown that groups of rats or mice often gain weight over the observation period of a study^36^, and we consider this model to be efficient for experimental gastric restriction. Not only was weight gain prevented in the restrict group, but weight was lost in 10 days; a total of 4% of the initial weight. The control and sham groups gained weight over the 10-day study period; 11% and 5.2%, respectively, of the initial weight gained.

Regarding the inflammatory cells, polymorphonuclear cells were enhanced in all groups compared to lymphomononuclear cells, which was significant between the groups, showing inflammation compared to the control group but not different from the sham group. This inflammatory response probably occurred due to the presence of the phytobezoar, which may also have contributed to a decrease in the supply of nutrients to the intestines. To evaluate the evolution of this inflammatory response, it may be necessary to increase the time of exposure to the phytobezoar.

Experimental studies have shown a reduction in height of the colonic glands in the colonic mucosa devoid of the fecal stream^26^. The authors suggest that a lack of SCFA supply and the oxidative stress in the colonic mucosa without a fecal stream are the main factors associated with colonic gland atrophy. In this study, we found that after 10 days of gastric restriction, the height of the crypts in general decreased compared to the sham and control groups. This has also occurred in experimental models of diversion colitis, where food restriction due to a phytobezoar probably led to the atrophy of the colonic glands. Interestingly, no differences were seen in the number of goblet cells. Controversies exist regarding the population of cells in colonic segments devoid of the fecal stream^37,38^. It is possible that the different results found in relation to the goblet cell population in the colonic mucosa restricting the regular supply of SCFAs may be related to the different exclusion times adopted in the experiment durations of different studies^22^.

The major function of intestinal goblet cells is the formation of mucus layers, which represent the first line of defense in the colonic epithelium^22,27^. Intestinal mucus layers secreted by goblet cells are rich in mucins, which provide the frontline defense of the host against endogenous and exogenous irritants and microbial attachment and invasion, but allow the transport of nutrients^24,25^. In most intestinal infections, the induction of goblet cells and mucin synthesis and secretion occur frequently during the acute phase. However, chronic infection results in the depletion of goblet cells^24,25,39^. Neutral mucins were enhanced even though their goblet cells did not increase, although the height of the crypt became higher. Neutral mucins represent the main subtype of mucins in the right colon, and the content of this type of mucins usually undergoes minor modifications in the colonic mucosa without a regular supply of feces^22^. SCFAs are crucial for intestinal health because they serve as the major energy substrates for colonocytes and have anti-inflammatory and anti-carcinogenic properties^40^. Furthermore, under germfree conditions, neutral mucins in the colon are higher^41^. The proportions between neutral and acid mucins in rats also occur in men and are usually constant in the colonic mucosa^42,43^. However, the tissue content of acidic mucins can be modified with food restriction, which should influence how the nourishment of the colonic mucosa interferes with the production and absorption of SCFAs^22,44^. This feature may be associated with intestinal barrier dysfunction^24,25,39,40,45,46^. The tissue content of total acid mucins was not increased. The proportion between sulfomucins and sialomucins is usually constant in the mucosa of normal colon tissue, but may undergo changes in various illnesses^41,46^.

One study showed that in the colonic mucosa without a fecal stream, the tissue content of sialomucins decreased, and this reduction was related to the time of fecal exclusion^27^_._ Gastric restriction substantially reduces the supply of fibers to the colon, thereby reducing the production of SCFAs^26^. This lower supply of SCFAs to the colonic mucosa, the main substrate for epithelial cells, causes less mucin production, as demonstrated by studies that measured the glycoprotein content in the colonic mucosa devoid of a fecal stream^22,27^. In this study, it is possible that we did not find modifications in the tissue content of sialomucins because a phytobezoar does not completely prevent the passage of SCFAs into the colon, thus maintaining a content of sialomucins similar to that of the sham group. Additionally, under germfree conditions, the ratio of neutral to acidic mucins in the colon is higher, and sulfomucins appear to increase at the expense of sialylated mucins^48,-51^. Similar to other authors who studied the tissue content of sialomucins in the colonic mucosa devoid of a fecal stream, we also found a lower expression of sialomucins and no difference in sulfomucins. It is likely that due to the incomplete absence of food, the acid mucins were not reshaped, but the goblet cells that express the sulfomucins were enhanced. This phenomenon could be triggered by the food restriction at the time. In general, trophism is conserved with the enhancement of neutral mucins. Collagen was enhanced in the restrict group, probably due to the inflammatory process triggered by gastric restriction.

To the best of our knowledge, no studies have quantified the expression patterns of acid and neutral mucins in an experimental model with gastric restriction.

Epithelial integrity, mucus production, and the presence and equilibrium of commensal bacteria interfere directly with the immunity of the intestinal barrier. The gastrointestinal epithelium is protected by a layer of mucus containing glycoprotein mucins secreted against the attack of digestive fluids, microorganisms, and toxins^52^. The integrity of the protective mucus layer is ensured by the rapid and massive secretion of goblet cells. Intestinal inflammation and injury result from a defective mucosal barrier, abnormal commensal bacteria, and defective host innate and adaptive immunitye^53,54^.

Overweight and obesity have reached epidemic levels in the United States and worldwide, affecting nearly three-fourths of adults in the United States^55-57^. Childhood obesity is one of the most serious global public health challenges of the 21st century, affecting every country in the world. In just 40 years, the number of school-age children and adolescents with obesity has risen more than 10-fold, from 11 million to 124 million (2016 estimates) (NCD Risk Factor Collaboration [NCD-RisC] 2017). In addition, in this same population, an estimated 216 million were classified as overweight but not obese in 2016 (NCD Risk Factor Collaboration [NCD-RisC] 2017).

The condition also affects younger children, with over 38 million children under the age of 5 living with overweight or obesity in 2017^58^. Bariatric surgery is considered for patients with severe obesity^57^. This study contributes to assessing the impact of weight loss strategies and to the effectiveness and safety of gastric restriction therapy, which has been increasing worldwide.

## Conclusions

The colon mucosa is affected by ten days of gastric restriction. In an animal model of inflammatory situations, dietary restriction can promote mucin synthesis, equilibrate the gut microbiota, and thus favor colonic protection and mucosal healing. Trophism is conserved with 10 days of gastric restriction.

## Footnotes

1 Research performed at the Laboratory of Descriptive and Topographic Anatomy Division (LIM 02), Laboratory of Surgical Physiopathology (LIM 62), and Center for Study and Research in Surgery (CEPEC), Department of Surgery, Faculty of Medicine, Universidade de São Paulo (FMUSP), Brazil.
